# The antidepressant fluoxetine (Prozac^®^) modulates estrogen signaling in the uterus and alters estrous cycles in mice

**DOI:** 10.1016/j.mce.2022.111783

**Published:** 2022-10-02

**Authors:** Rafael R. Domingues, Milo C. Wiltbank, Laura L. Hernandez

**Affiliations:** a Department of Animal and Dairy Sciences, University of Wisconsin-Madison, Madison, WI, USA; b Endocrinology and Reproductive Physiology Program, University of Wisconsin-Madison, Madison, WI, USA

**Keywords:** Anovulation, Estrogen signaling, Endocrine disruption, Fluoxetine

## Abstract

Selective serotonin reuptake inhibitors (SSRI) are the most used antidepressants. However, up to 80% of women taking SSRI suffer from sexual dysfunction. We investigated the effects of fluoxetine (Prozac^®^) (low and high dose, n = 6–7/group) on reproductive function and the regulation of the estrous cycle. All mice treated with high dose of fluoxetine had interruption of estrous cycles within a few days after onset of treatment. When treated for 14 days, mice in the high dose group had fewer CL, often lack of any CL, and antral follicles. Uterine expression of estrogen receptor alpha, G-protein coupled estrogen receptor, and steroidogenesis enzymes were upregulated in the high dose group. Nevertheless, decreased expression of connexin 43 and alkaline phosphatase and increased expression of insulin-like growth factor-binding protein 3 and monoamine oxidase A are consistent with decreased estrogen signaling and the decreased uterine weight. Taken together, fluoxetine modulates estrogen synthesis/signaling and dysregulates estrous cycles.

## 1. Introduction

About 6% of adolescents aged 12–19 have used psychotropic medications and 17.7% of women older than 18 years of age have used antidepressants (Brody DJ, 2020). Among these drugs, selective serotonin reuptake inhibitors (SSRI) are the most commonly prescribed. Although SSRI improve mental health, multiple studies have reported side effects of SSRI including reproductive issues (Alwan et al., 2016; Bandoli et al., 2020; Domingues et al., 2022a; Belik, 2008; Zhao et al., 2018). Indeed, the effects of SSRI on pregnancy outcomes have been extensively explored in the past decade and appear to be associated with modulation of serotonin signaling (Domingues et al., 2022a, 2022b; Zhao et al., 2018; Velasquez et al., 2013; Tran and Robb, 2015; Hensley and Nurnberg, 2002; Jing and Straw-Wilson, 2016; Steiner et al., 1997). However, the effects of SSRI on reproductive function in a nonpregnant state are poorly understood (Mondal et al., 2013).

The main biological target of SSRI is serotonin transporter (SERT) (DeVane, 1999). Accordingly, SSRI inhibition of SERT modulates serotonin availability and signaling in the brain (antidepressant effect) and in the periphery (possible side effects) (Blardi et al., 2002). Indeed, SSRI inhibition of platelet SERT leads to increased free (plasma) concentrations of serotonin (Blardi et al., 2002). However, SSRIs seem to also modulate synthesis and signaling of other hormones (Lupu et al., 2017; Muller et al., 2012; Papakostas et al., 2006). All clinically available SSRI have endocrine disrupting effects on steroid hormone synthesis in vitro (Hansen et al., 2017; Jacobsen et al., 2015). Fluoxetine and sertraline stimulate aromatase activity leading to increased estradiol synthesis. Additionally, SSRI use has been associated with hyperprolactinemia in women and rodents (Papakostas et al., 2006; Peterson, 2001). However, the endocrine disruption impacts of SSRI on the regulation of reproductive cycles are poorly understood.

In humans, SSRI cause sexual dysfunction and in rodent models SSRI affect sexual behavior (Jing and Straw-Wilson, 2016; Mondal et al., 2013; Maswood et al., 2008). In addition to the increased rates of women suffering from depression compared to men, and consequently undergoing SSRI treatment, women display more severe SSRI-induced sexual dysfunction (Hensley and Nurnberg, 2002). It has been reported that up to 80% of women taking SSRI display some sort of sexual dysfunction. Because sexual dysfunction is often due to endocrine influence (Carosa et al., 2020), delineating the mechanisms underpinning the effects of SSRI on endocrine, ovarian, and uterine function will be critical for defining the role of SSRI on sexual dysfunction and to mitigate these effects without affecting the beneficial antidepressant effects.

Due to the potential endocrine disrupting effects of SSRI on women’s reproductive function, we aimed to delineate the effect of fluoxetine on reproductive function and the regulation of estrous cycles in sexually mature mice. We hypothesized that fluoxetine modulates estrogen synthesis/signaling and dysregulates estrous cycles.

## 2. Materials and methods

### 2.1. Animals

All experimental procedures were approved by the Research Animal Care and Use Committee at the University of Wisconsin-Madison and were performed under protocol number A005789-A01. Mice were individually housed in a controlled environmental facility for biological research in the Animal and Dairy Sciences Department vivarium at the University of Wisconsin-Madison. Animal facility was maintained at a temperature of 25 °C and a humidity of 50%–60%, with a 12:12 h light-dark cycle with *ad libitum* water and food (LabDiet 5015, TestDiet, Richmond, IN). Wild-type C57BL/6J mice (N = 20) were obtained from Jackson Laboratories (stock # 000664, Jackson Laboratories, Bar Harbor, ME).

### 2.2. Experimental design

Beginning at six-weeks of age, stage of estrous cycle of virgin female mice was determined daily for 20 days to establish normal estrous cyclicity in all animals. After establishing normal cyclicity in all mice, daily intraperitoneal injection began (day 0) on randon days of the estrous cycle. Mice were randomly allocated to a vehicle (saline, n = 6), low dose fluoxetine (2 mg/kg/d, n = 7; fluoxetine hydrochloride, F312; Sigma-Aldrich, St. Louis, MO), and high dose fluoxetine (20 mg/kg/d, n = 7) treatment groups. Determination of estrous cycles and treatments continued daily until day 14. Mice were weighed daily throughout the experimental period.

We have previously used these doses of fluoxetine in mice (Domingues et al., 2022a). The low dose (2 mg/kg/d) results in systemic concentration similar to that of humans taking fluoxetine. The high dose (20 mg/kg/d) results in greater systemic concentrations although it is commonly used in mice studies (Walia and Gilhotra, 2017; Ma et al., 2021).

### 2.3. Determination of phases of estrous cycle

To determine stage of estrous cycles, vaginal lavage was performed daily as described (Cora et al., 2015) between 9 and 10 a.m. by the same technician throughout the experimental period. Slides were stained with Wright-Giemsa (Hema3 Stat Pack, Fisherbrand, Pittsburgh, PA, USA) and observed using a light microscope.

### 2.4. Blood and tissue collection

Mice were euthanized approximately 6 h after the last treatment (day 14) with carbon dioxide followed by cervical dislocation. Cardiac blood was collected immediately after euthanasia. Uterus was excised and weighed. One uterine horn along with the ovaries were fixed in 4% paraformaldehyde overnight and stored in 70% ethanol until histological processing. Histology samples were embedded in paraffin, sectioned into 8 μm sections, stained with conventional hematoxylin-eosin and observed by light microscopy for image collection and analyzed qualitatively by a single technician unaware of treatment group. The other uterine horn was snap frozen in liquid nitrogen and stored at 80 °C and used for evaluation of gene expression.

### 2.5. Extraction of RNA, complementary DNA, and quantitative PCR

Extraction of RNA was performed with Trizol reagent (Invitrogen, CA, USA) as described by the manufacturer and quantified by spectrometry with a NanoDrop 2000 spectrophotometer (Thermo Scientific, USA). Complementary DNA (cDNA) was synthesized using the High-Capacity cDNA Reverse Transcription Kit (Applied Biosystems, CA, USA) as described by the manufacturer using 1 μg of total RNA. The cDNA was used directly for quantitative real-time PCR (qRT-PCR). The qRT-PCR reactions were carried out on an CFX Connect Real-Time PCR system (Bio-Rad Life Science, CA, USA) using a master mix that contained a total volume of 10.5 μL per tube consisting of 6.25 μL of SsoFast EvaGreen Supermix (Bio-Rad Laboratories Inc., CA, USA), 3.25 μL of nuclease-free water, and 0.5 μL of forward and reverse primers (10 μM). Two μL of cDNA at a 1:5 dilution was added to the master mix for a total reaction volume of 12.5 μL. All samples were evaluated in duplicate. The reactions were initiated with preincubation at 95 °C for 3 min followed by 42 cycles of denaturation (95 °C for 10 s) and annealing and extension (60 °C for 30 s).

The primer sequences for targeted genes (Table 1) were synthesized by Integrated DNA Technologies Inc. (CA, USA) using sequences reported in previous studies or designed by our laboratory. Efficiencies of qRT-PCR for amplification of targeted genes were determined in our laboratory and ranged from 95% to 107%. The amplification data obtained from the qRT-PCR were the cycle threshold (Ct) for each mRNA and these was used to calculate the mRNA relative abundance of each sample by the 2^−ΔΔCt^ method (Livak and Schmittgen, 2001) using vehicle group as baseline and the geometric mean of the housekeeping genes 36b4 and Hrpt1.

### 2.6. Statistical analysis

All statistical analyses were performed with SAS (version 9.4; SAS Institute Inc., Cary, North Carolina, USA). Data were analyzed with the PROC MIXED procedure using one-way ANOVA and two-way ANOVA for repeated measures. Tukey HSD was used for post hoc comparisons. Residuals with deviations from assumptions of normality and/or homogeneity of variance were transformed into square root, logarithms, or ranks. A probability of ≤0.05 indicated a difference was significant and a probability between >0.05 and ≤0.1 indicated tendency for significance. Data are presented as the mean ± standard error of mean (SEM).

## 3. Results and discussion

Understanding the endocrine disruptive effects of psychotropic medications and their implications on reproductive function is critical so that patients and physicians can make informed decisions when developing treatment plans. Furthermore, understanding the side effects of currently prescribed drugs is essential for the development of new, improved treatments with fewer side effects.

**Fig. 1.**
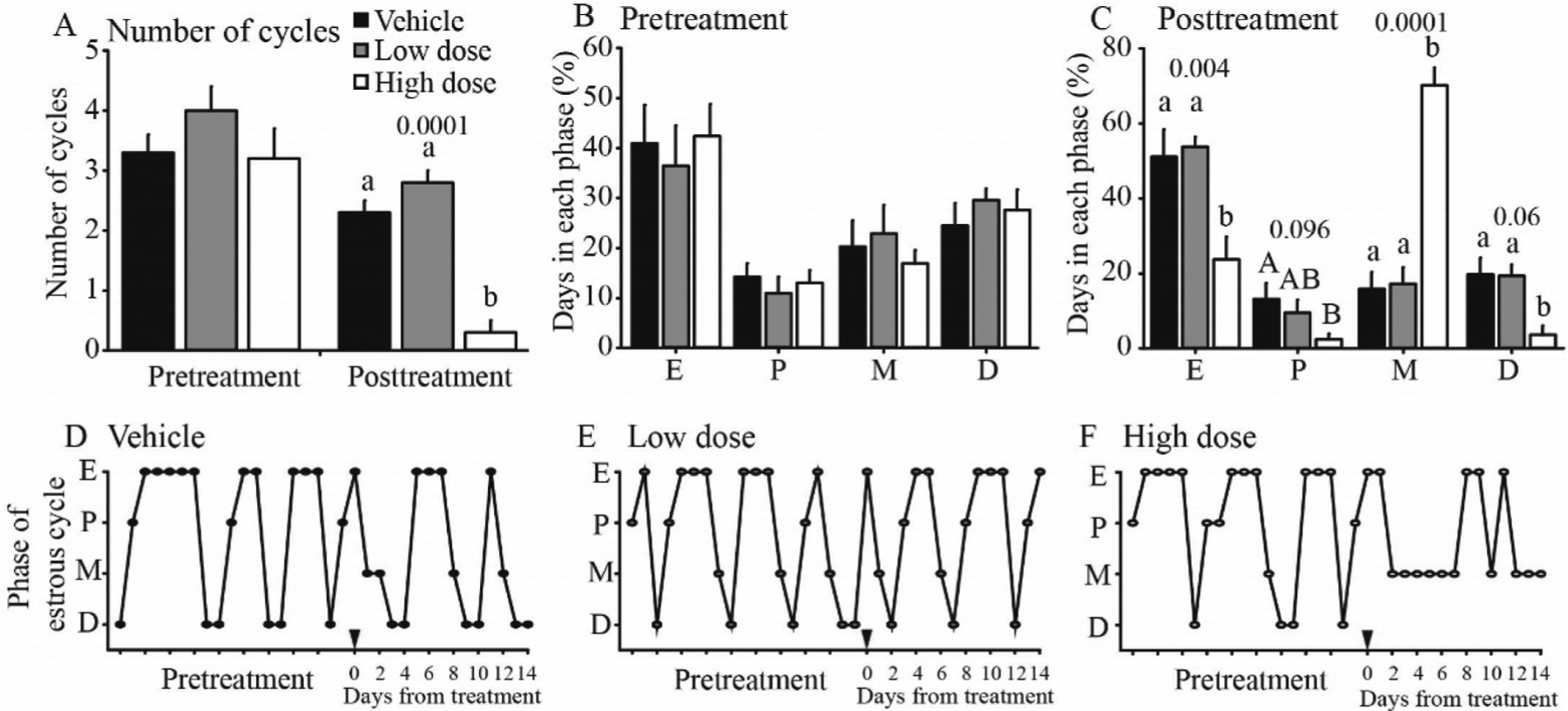
Estrous cycles before and after onset of treatment (day 0) for mice treated with vehicle (saline), low dose fluoxetine (2 mg/kg/d), and high dose fluoxetine (20 mg/kg/d) for 14 days. (A) Number of estrous cycles. Percentage of period in each stage of the estrous cycle during pretreatment (B) and after onset of treatment (posttreatment; C). Estrous cycle pattern in a representative mouse during experimental period for vehicle (D), low dose (E), and high dose (F). E, estrus; M, metestrus; D, diestrus; P, proestrus. abc, indicate significant difference among groups. ABC, indicate tendency for significant difference among groups.

To determine the effect of fluoxetine on estrous cyclicity, we examined daily vaginal smears of sexually mature mice. Before onset of treatment, all mice had normal estrous cycles (Fig. 1). After treatment onset, vehicle and low dose groups continued to cycle regularly while estrous cycles were dramatically reduced in mice receiving the high dose of fluoxetine. All mice receiving high doses of fluoxetine had irregular estrous cycles after treatment onset. Vaginal cytology of mice receiving high doses of fluoxetine presented with mixed cell types with predominance of leukocytes and some anucleated keratinized epithelial cells, which is characteristic of metestrus (Cora et al., 2015; Ajayi and Akhigbe, 2020) and typically observed in rodents chronically treated with estradiol or exposed to estrogenic endocrine disrupting compounds (Sano et al., 2020).

**Fig. 2.**
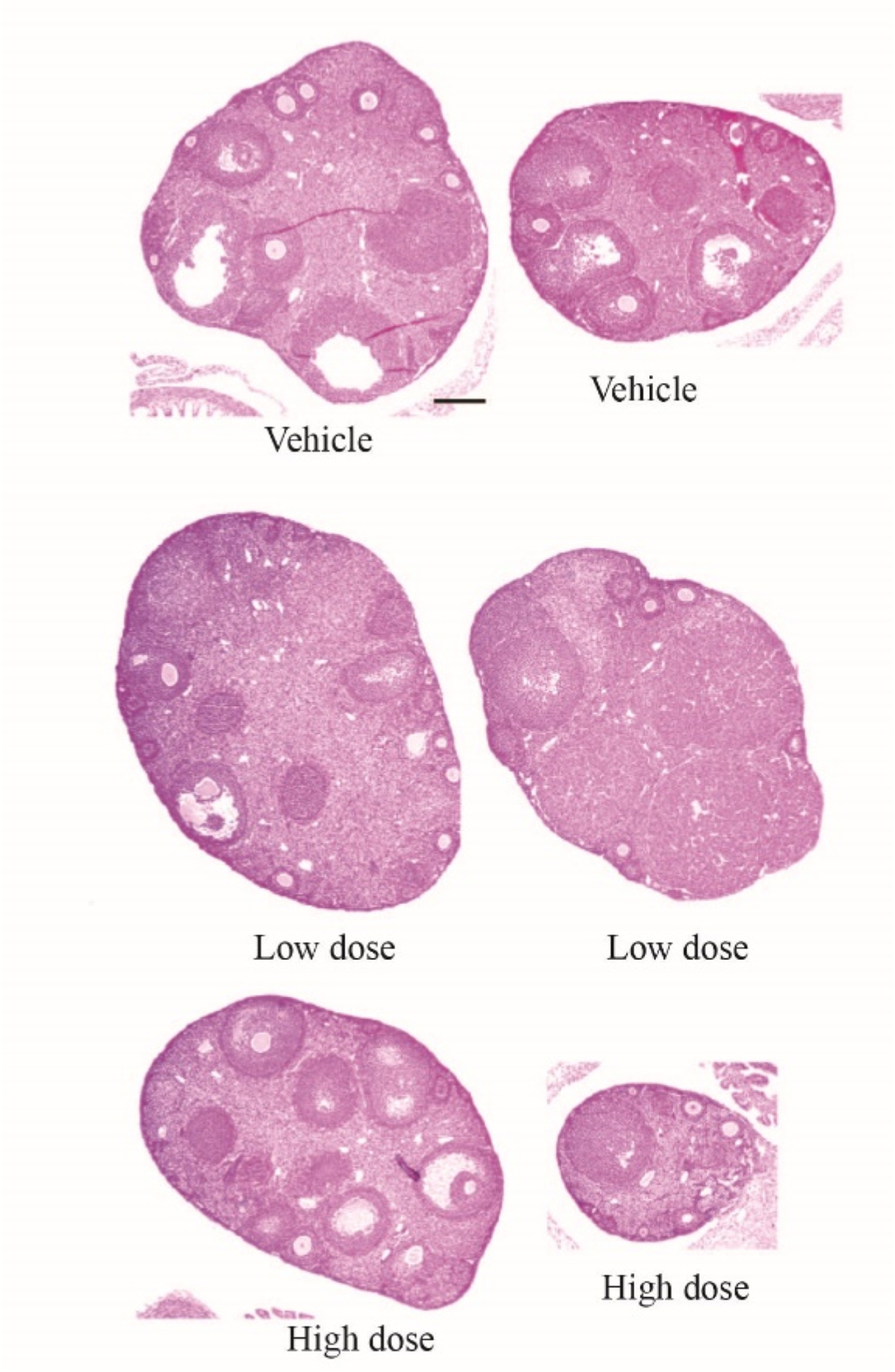
Ovarian histology of two representative mice treated for 14 days with vehicle (saline), low dose fluoxetine (2 mg/kg/d), and high dose fluoxetine (20 mg/kg/d). Images were taken at 4x magnification (scale bar: 2 mm).

Fewer CL, often lack of any CL, and large antral follicles were observed in the ovaries from mice in the high dose group (Fig. 2). In previous studies, fluoxetine treatment caused follicle and oocyte abnormalities, increased number of atretic follicles, and decreased number of ovulated oocytes (Romero-Reyes et al., 2016; Achary and Rohini, 2021; Mansoriyan et al., 2018). Furthermore, fluoxetine increased the number of small and medium preantral follicles which might be associated with decreased follicle development beyond that stage resulting in decreased ovulation and subsequent development of CL, as observed in the present study (Romero-Reyes et al., 2016). Similarly, fluoxetine reduced ovulation in Balb/C mice (Mansoriyan et al., 2018) and rabbits (Pavlicev et al., 2019). The ovarian findings reported in the present and previous studies are consistent with fluoxetine-induced disruption of ovarian function and interruption of estrous cycles directing some caution for women and girls of reproductive age.

Fluoxetine and sertraline, the most commonly used SSRI (Bandoli et al., 2020), increase estradiol synthesis in vitro and fluoxetine has estrogenic effects in vivo (Muller et al., 2012; Hansen et al., 2017; Jacobsen et al., 2015). Interestingly, in vivo short-term fluoxetine treatment seems to increase systemic concentrations of estrogen (Muller et al., 2012) whereas long-term treatment appears to reduce it (Taylor et al., 2004; Mennigen et al., 2017). The fluoxetine-induced increase in concentrations of estradiol may affect hypothalamic secretion of GnRH and pituitary secretion of FSH/LH (Kasturi et al., 2013). Alternatively, the fluoxetine-induced increase in neuronal serotonin signaling may directly alter GnRH and LH pulses (Wada et al., 2006; Johns et al., 1982; Bhattarai et al., 2014). Previous studies have shown that fluoxetine treatment rapidly decreases LH pulses in rats (Rasmussen et al., 1981) and four weeks of treatment decreases estradiol and FSH. The effects of fluoxetine on the hypothalamic-pituitary-gonadal axis (Ebrahimian et al., 2014) may lead to decreased follicle progression into preovulatory stages resulting in decreased ovulation rate as observed in the present and previous studies (Romero-Reyes et al., 2016; Mansoriyan et al., 2018; Pavlicev et al., 2019). Noteworthy, chronic estrogen treatment alters estrous cycles causing decreased number of cycles, absence of CL, and ovary atrophy (Kasturi et al., 2013) consistent with findings in the present study. Decreased follicle development is consistent with decreased concentrations of estradiol as observed after long-term fluoxetine treatment since ovarian follicles are the main source of circulating estradiol. Although estradiol concentrations were not measured in the present study because of the confounding effect of euthanasia/blood collection on different days of the estrous cycles, the ovarian and uterine findings in the present study are consistent with decreased systemic concentrations of estradiol.

**Fig. 3.**
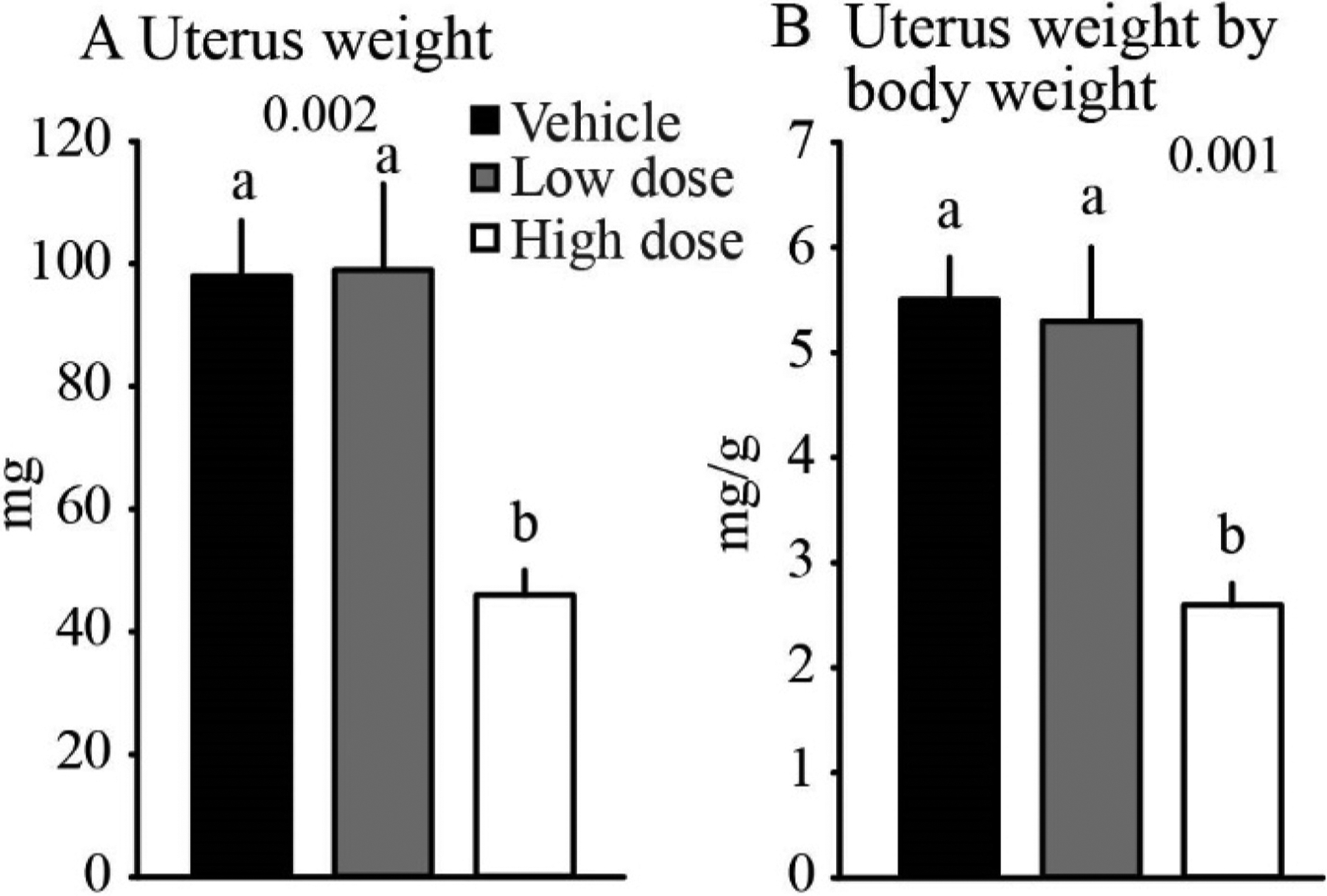
Uterus weight (A) and uterus weight by body weight (B) for mice treated for 14 days with: vehicle (saline), low dose fluoxetine (2 mg/kg/d), and high dose fluoxetine (20 mg/kg/d). abc, indicate significant difference among groups.

Uterine weight was reduced (P = 0.002) by 53% in the high dose group (45.7 ± 3.7 mg) compared to the vehicle (98.0 ± 9.1 mg) and low dose (98.6 ± 14.2 mg) groups (Fig. 3). Furthermore, uterine weight adjusted to body weight was reduced (P = 0.0014) in the high dose group (2.6 ± 0.2) compared to the vehicle (5.5 ± 0.4) and low dose (5.3 ± 0.7) groups. The reduced uterine weight in the high dose group is consistent with decreased concentrations of estradiol and the interruption of estrous cycles. No major histological abnormalities were observed in the uterus.

**Fig. 4.**
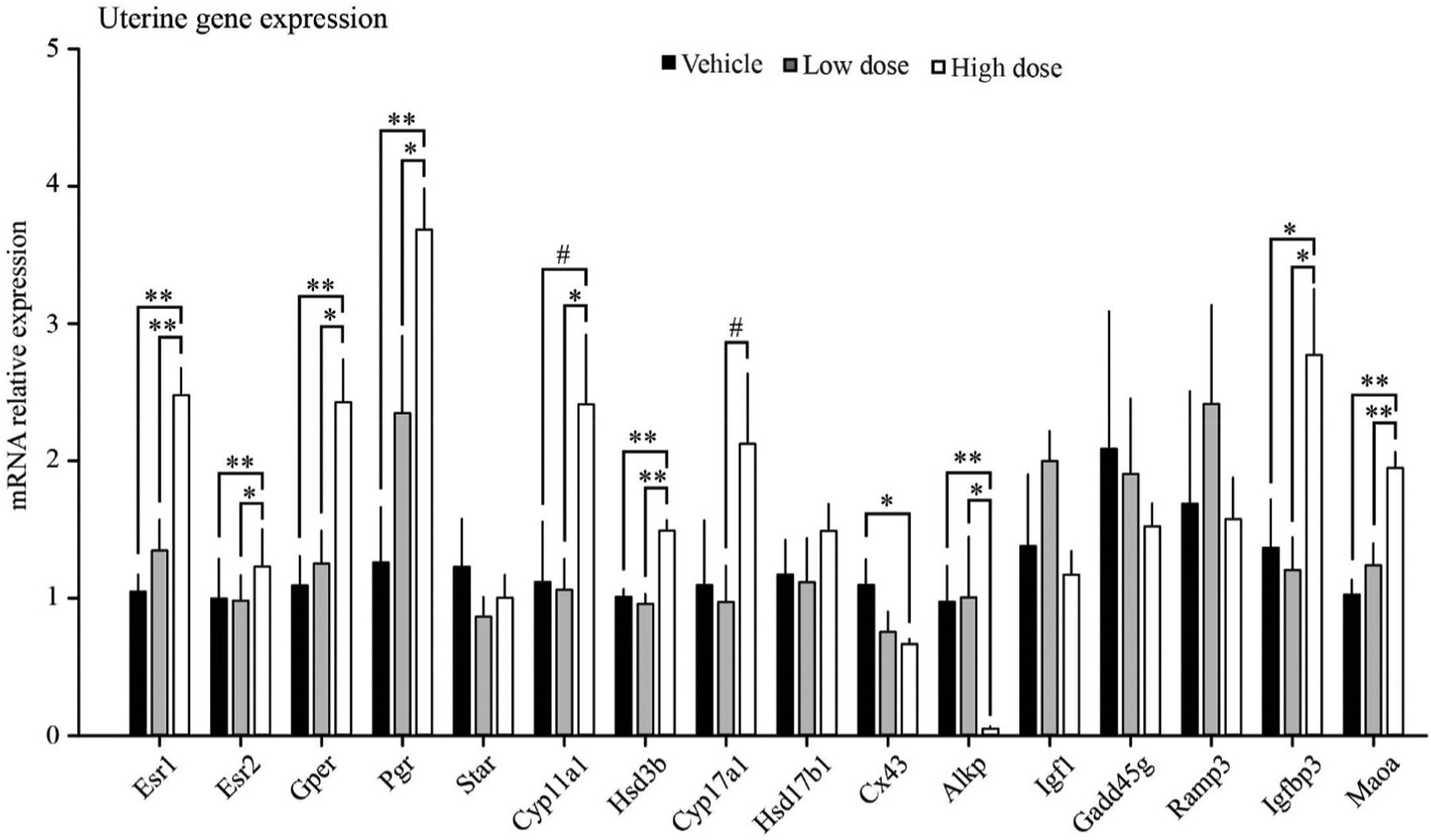
Uterine gene expression for mice treated for 14 days with: vehicle (saline), low dose fluoxetine (2 mg/kg/d), and high dose fluoxetine (20 mg/kg/d). ** indicate P-value < 0.01; * indicate P-value < 0.05; # indicate P-value < 0.1. ABC, indicate tendency for significant difference among groups.

The high dose of fluoxetine increased uterine expression of estradiol receptor alpha (Esr1; 2.4-fold), G-protein coupled estrogen receptor (Gper; 2.2-fold), and steroidogenic enzymes (Hsd3b [1.5-fold], Cyp11a1 [2.1-fold]) (Fig. 4). Expression of aromatase in the uterus was too low to be reliably analyzed (qRT-PCR Ct > 40). Although the uterus is not commonly thought as a steroidogenic organ, it does synthesize estrogen under different stages of the estrous cycle, pregnancy, and in some disease conditions (Das et al., 2009; Tseng, 1984; Delvoux et al., 2009). In the present study, the increased uterine expression of estrogen receptors and steroidogenic enzymes are inconsistent with decreased uterine weight since estrogen signaling typically increases uterine cell proliferation and uterine weight (Evans et al., 1941). Nevertheless, the decreased expression of connexin 43 (Cx43; ~39% reduction) and alkaline phosphatase (Alkp; ~95% reduction), major regulators/markers of uterine stromal differentiation, are consistent with decreased estrogen signaling and decreased uterine weight (Das et al., 2009). Additionally, classic estrogen responsive genes were not different among groups (Igf1, Gadd45g, Ramp3) (Groothuis et al., 2007; Labrie et al., 2012) while expression of genes typically downregulated by estrogens were increased (Igfbp3 [2-fold] and Maoa [1.9-fold]) (Huynh and Pollak, 1994; Holzbauer and Youdim, 1973; Collins and Southgate, 1970). Furthermore, Igfbp3 had been reported to be inversely related to uterine weight (Huynh and Pollak, 1994) as observed in the present study. Taken together, uterine gene expression along with uterine weight suggest decreased estrogen signaling. Further studies are needed to confirm the effects of fluoxetine on uterine function after short and long-term exposure and implications for reproductive health and pregnancy.

**Fig. 5.**
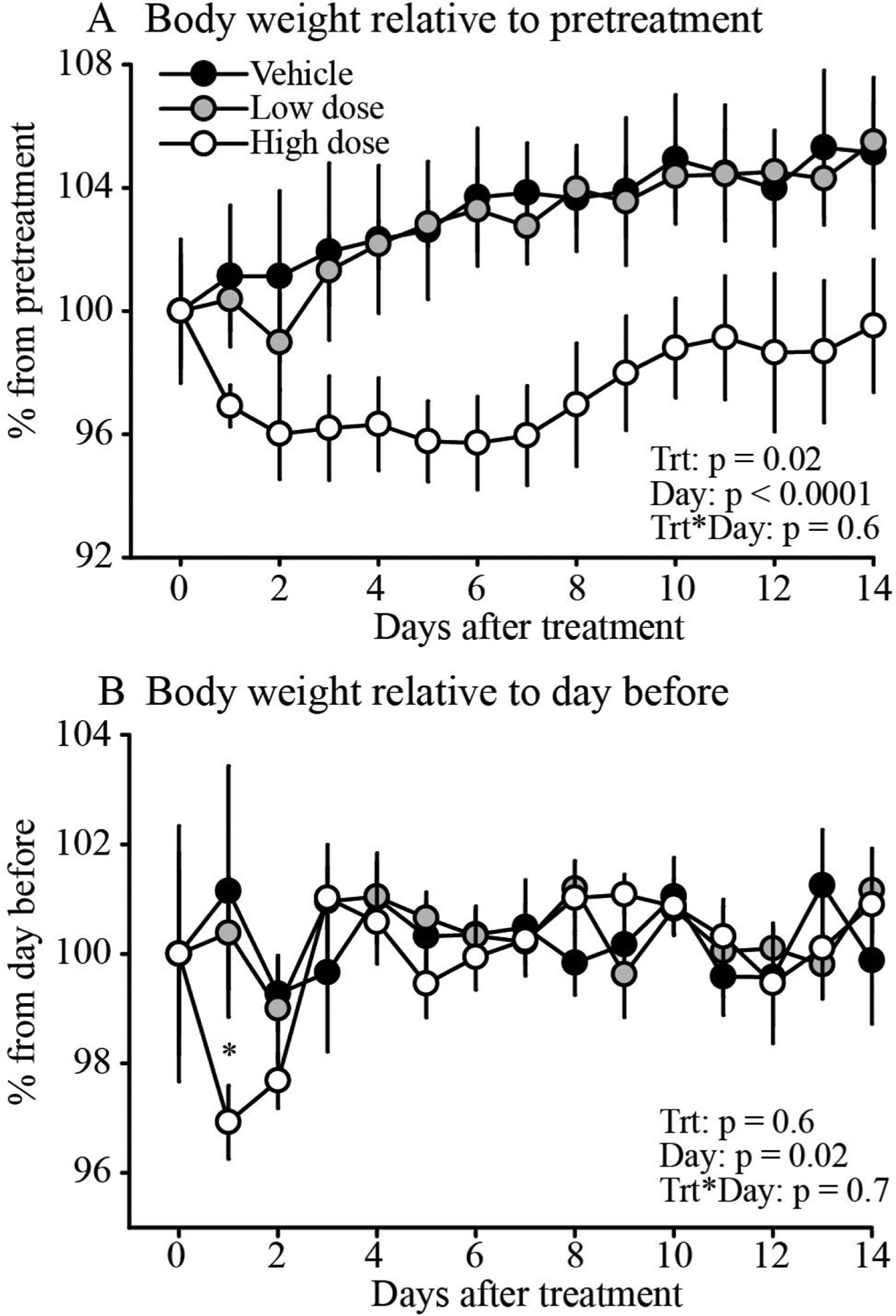
Body weight for mice treated for 14 days with: vehicle (saline), low dose fluoxetine (2 mg/kg/d), and high dose fluoxetine (20 mg/kg/d). Weight change relative to pretreatment (A). Weight change relative to day before (B). *, indicate significant difference among groups based on separate analysis for day 1.

Mouse body weights were not different (P = 0.67) among groups before the onset of treatment (overall mean body weight was 17.3 ± 0.2 g). During the 14-day treatment regimen, body weight relative to the pretreatment period was reduced in the high dose fluoxetine group compared to vehicle and low doses groups (Fig. 5). Nevertheless, daily weight change relative to day before was not different among groups when evaluated during the overall treatment period. However, after the first treatment the vehicle and low dose groups gained weight (1.1 ± 2.0 and 0.4 ± 1.0%, respectively) while the high dose fluoxetine group lost weight (3.1 ± 0.7%; P = 0.02). Collectively, the high dose of fluoxetine caused only a transient weight loss after the first day of treatment. Similarly, we have previously observed that the high dose of fluoxetine caused transient weight loss in pregnant mice (Domingues et al., 2022a). Furthermore, other studies also reported reduced weight gain in mice treated with fluoxetine, particularly at higher doses (Maswood et al., 2008; Uphouse et al., 2006; Muller et al., 2013; Aggarwal et al., 2016). A previous study suggested that the fluoxetine-induced anovulation in Fishers rats was due to decreased food consumption and weight loss caused by fluoxetine (Uphouse et al., 2006). However, in another study from the same laboratory using Sprague Dawley rats the reproductive effects of fluoxetine were only mild with longer estrous cycles in 40% of animals but only after 10 days of treatment and no anovulation (Maswood et al., 2008). Previous research in our laboratory did not observe a significant decrease in feed consumption in C57Bl/6J mice treated with fluoxetine and, similar to this study, weight loss only lasted for a day (Hernandez, unpublished). Therefore, it is unlikely that interruption of estrous cycles in the present study was due to decreased feed intake/weight loss.

Another important effect of SSRI on reproduction is its modulation of sexual behavior in both humans and rodent models. Clinical evidence of a role for SSRI on modulation of sexual behavior is its use to delay ejaculation in men with premature ejaculation and in men with erectile failure (Powersmith, 1994; Machale and Phanjoo, 1994). In women, SSRI use has been associated with decreased sexual desire, excitement, and delayed orgasm (Higgins et al., 2010). In rodent models, medication that increase serotonin concentrations and signaling in the brain, such as SSRI, negatively affect female sexual behavior (Uphouse, 2014). Accordingly, fluoxetine has an acute (within 30 min) dose-dependent reduction in lordosis response to mounting in intact and in ovariectomized, hormone primed rats suggesting a central effect of the drug on sexual behavior (Miryala et al., 2013). Taken together, the effects of SSRI on reproductive function go beyond physiological changes in reproductive organs but also affect sexual behavior which may extend its compromise on reproductive efficiency and women try to become pregnant.

## 4. Conclusions

In conclusion, we report that fluoxetine treatment results in the disruption of ovarian and uterine function with consequent interruption of estrous cycles. Treatment with the high dose of fluoxetine for 14 days caused anovulation and altered ovarian morphology. Additionally, uterine weight and gene expression were altered. Taken together, the interruption of estrous cycles along with ovarian and uterine changes suggest lack of follicle development/ovulation and decreased estrogen signaling. This is a critical finding given the number of adolescent and adult women prescribed SSRI and provides a framework for other research exploring the endocrine disrupting effects of SSRI.

## Figures and Tables

**Table 1:** Primer sequences for quantitative real time PCR.

Gene	Primer sequence	GenBanker ID
36b4	F: CCTATAAAAGGCACACGCGG	NM_007475.5
	R: ACGCGGGGTTTAAAGACGAT	
Hrpt1	F: GCTTCCTCCTCAGACCGCTT	NM_013556
	R: ATCGCTAATCACGACGCTGG	
Esr1	F: TGATGCCAGGAGAGGCCAATGC	NM_007956.4
	R: TGTCGCCCAGAGACTGCCTTCTT	
Esr2	F: GCCAGCCCTGTTACTAGTCCAA	NM_207707.1
	R: CAGACGGCGCAGAAGTGA	
Gper	F: CTGCACGAGCGGTACTACGA	XM_036165593.1
	R: CAGATGAGGCCACAGCTCAG	
Pgr	F: TATGGCGTGCTTACCTGTGG	NM_008829.2
	R: TGCCAGCCTGACAACACTT	
StAR	F: CTGCAGGACTCAGGACCTTG	NM_011485.5
	R: ACACAGCTTGAACGTAGCGA	
Cyp11a1	F: TGCTCTGCAAAGCCGAATAC	NM_001346787.1
	R: TGCTCTGCAAAGCCGAATAC	
Hsd3b	F: GTGCGCCCTGGGACTTACTA	NM_133943.2
	R: ACCAGGTATACCAGTGTTGGC	
Cyp17a1	F: GGCCCAAGTCAAAGACACCT	NM_007809.3
	R: CGTCTGGGGAGAAACGGTAG	
Hsd17b1	F: ATGTGCTTGGGACCATTCGG	NM_010475.2
	R: GTGGAATGGCAGTCCCATCA	
Cx43	F: ACGCTTTTACGAGGTATCAGCA	NM_010288.3
	R: GTCTGCTGCTGTTGGGTACT	
Alkp	F: CTACGCACCCTGTTCTGAGG	XM_006538498.4F
	R: GACCTCTCCCTTGAGTGTGG	
Igf1	F: GCTCTTCAGTTCGTGTGTGGAC	NM_001111276.1
	R: AGCCTGTGGGCTTGTTGAAGTA	
Gadd45g	F: AGTCCTGAATGTGGACCCTGACA	NM_011817.2
	R: GCAGAACGCCTGAATCAACGTG	
Ramp3	F: GTTGCTGCTTTGTGGTGAGTGT	NM_019511
	R: AGACAGCCACCTTCTGCATCAT	
Igfbp3	F: TGTGTGGACAAGTATGGGCAGC	NM_008343.2
	R: TGAGCTCCATATTATGTGGCACGG	
Maoa	F: ACAGCAACACAGTGGAGTGG	NM_173740.3
	R: GGAACATCCTTGGACTCAGG	

## Data Availability

Data will be made available on request.
